# Primed for Discovery: Atomic-Resolution Cryo-EM Structure of a Reovirus Entry Intermediate

**DOI:** 10.3390/v2061340

**Published:** 2010-06-15

**Authors:** Shane D. Trask, Kristen M. Guglielmi, John T. Patton

**Affiliations:** Laboratory of Infectious Diseases, National Institute of Allergy and Infectious Diseases, National Institutes of Health, Bethesda, MD 20892, USA; E-Mails: trasks@niaid.nih.gov (S.D.T.); guglielmikm@niaid.nih.gov (K.M.G.)

**Keywords:** aquareovirus, orthoreovirus, Reoviridae, electron cryomicroscopy, nonenveloped virus, virus entry, myristoyl, autoproteolysis

## Abstract

A recently solved structure of the aquareovirus virion (Zhang, X; Jin, L.; Fang, Q; Hui, W.H.; Zhou Z.H. 3.3 Å Cryo-EM Structure of a Nonenveloped Virus Reveals a Priming Mechanism for Cell Entry. *Cell* **2010**, *141*, 472–482 [1]) provides new insights into the order of entry events, as well as confirming and refining several aspects of the entry mechanism, for aquareovirus and the related orthoreovirus. In particular, the structure provides evidence of a defined order for the progressive proteolytic cleavages of myristoylated penetration protein VP5 that prime the virion for membrane penetration. These observations reinforce the concept that, much like enveloped viruses, nonenveloped virions often undergo priming events that lead to a meta-stable state, preparing the virus for membrane penetration under the appropriate circumstances. In addition, this and other recent studies highlight the increasing power of electron cryomicroscopy to analyze large, geometrically regular structures, such as icosahedral viruses, at atomic resolution.

## 1. Introduction

Electron cryomicroscopy (cryo-EM) has been a stalwart tool for virologists, yielding reconstructed virion structures for more than 20 years. Resolution has steadily improved, from initial reconstructions at greater than 20 Å to current ones that approach the resolutions seen in X-ray crystallographic structures (commonly ∼3–5 Å). At this scale it is possible to observe protein sidechains and to build accurate models directly from the cryo-EM data. One such structure, of the primed aquareovirus virion at 3.3 Å resolution, was recently reported in *Cell* [1]. The entry mechanism of reoviruses (used here to refer collectively to orthoreovirus and aquareovirus) has been an area of active inquiry for decades. Numerous structures of virus particles and isolated capsid proteins have been reported, and a wealth of biochemical data have been generated to describe the entry process of reovirus. Yet, by simply attaining a high-resolution reconstruction of the aquareovirus virion, Zhang and colleagues have uncovered new information about the order of events during reovirus membrane penetration. Recent reports of high-resolution cryo-EM structures of other viruses have echoed this phenomenon: By pushing cryo-EM reconstructions into near atomic-level resolution, we can gain new insights into the biology of viruses, even those with previously analyzed structures. The technical advances that permit this level of analysis include both more powerful instrumentation and new analysis software, but are ultimately limited by the regularity of the specimen.

## 2. The Mechanism of Reovirus Membrane Penetration

Reoviruses, and other members of the *Reoviridae* have multi-layered icosahedral capsids. The inner capsid particle is the “payload” that is delivered to the cell cytoplasm during infection, where it becomes transcriptionally active and initiates a round of replication. The outer capsid of the double-layered reovirus virion is a layer of hetero-hexamers comprised of three copies each of a “protection” protein (σ3 for orthoreovirus; VP7 for aquareovirus) and a “penetration” protein (μ1 for orthoreovirus; VP5 for aquareovirus). A previous crystal structure of the orthoreovirus μ1 and σ3 hetero-hexamer revealed that the μ1 subunits are Z-shaped and intertwine to form a trimer, while three σ3 molecules decorate the top of the μ1 trimer [2]. Cryo-EM reconstructions—including the current study—confirmed that the aquareovirus VP7 and VP5 hetero-hexamer is structurally very similar to that of orthoreovirus [1,3]. Orthoreovirus μ1 has an N-terminal myristate moiety, which is involved in membrane penetration. The 3.3 Å aquareovirus reconstruction verified that the covalently linked myristoyl group tucks into a hydrophobic pocket at the base of each VP5 monomer where it remains until it is released during the membrane penetration event [1,2].

The reovirus outer capsid undergoes an ordered disassembly that is triggered by several proteolysis events, ultimately leading to the release of hydrophobic fragments of the penetration protein and viral membrane penetration. The first proteolytic event leading to membrane penetration is the cleavage and removal of σ3/VP7 by the cathepsin family of cysteine proteases during endocytosis (or *in vitro* with α-chymotrypsin) [4]. Removal of σ3/VP7 results in an infectious sub-viral particle, or ISVP; it is this particle that Zhang and colleagues have analyzed. The ISVP is thought to be a meta-stable intermediate that is primed for membrane penetration, akin to the pre-fusion state of an enveloped virus fusion protein. Due to its meta-stable conformation, cryo-EM, rather than X-ray crystallography, is an ideal technique to analyze the structure of the ISVP, as it allows data collection quickly after sample preparation and in a chosen buffer.

The penetration protein, othoreovirus μ1, is the target of a second set of proteolysis events. An autocatalytic cleavage separates the myristoylated N-terminal peptide, μ1N, from the remainder of the protein [5,6]. Additional proteolysis events near the C-terminus separate the remainder of μ1 into fragments known as δ and ϕ. It is thought that μ1N and ϕ are released from the particle and act to form pores in membranes and recruit the reovirus core to these pores [7,8]. The high-resolution aquareovirus ISVP reconstruction unambiguously shows that autoproteolysis of μ1 homologue VP5, but not the δ-ϕ cleavage, occurs during the conversion from intact virion to ISVP [1]. There is an 11.7 Å gap that separates VP5 residue Asn42 from residue Pro43, which is obvious at 3.3 Å resolution, but may have been overlooked in lower-resolution structures. This finding clarifies confusion in the field regarding the order of cleavage events; it was previously suspected that μ1/VP5 autocatalytic cleavage and δ-ϕ cleavage occur concurrently, but much later during membrane penetration (after ISVP formation) [2]. The current observation that VP5 is also cleaved upon VP7 removal suggests that removal of the aquareovirus VP7 is coupled to a conformational change in the penetration protein that permits autoproteolysis. Consistent with this interpretation, by comparing the structure of the primed aquareovirus VP5 in the ISVP to the crystal structure of “unprimed” orthoreovirus μ1, Zhang and colleagues have identified a subtle conformational change that may trigger autoproteolysis of the virion-associated μ1/VP5 following removal of σ3/VP7 [1].

During entry, the ISVP is triggered to become an ISVP*, a particle in which dramatic conformational rearrangements of μ1 that increase the hydrophobic character of the virion are thought to have occurred [9]. This state most likely represents a high-energy intermediate, akin to the extended intermediate of an enveloped virus fusion protein that leads directly to a membrane fusion (penetration) event. Due to the unstable and transient nature of the ISVP*, little is known about the structure and organization of μ1 protein fragments.

**Figure 1:**
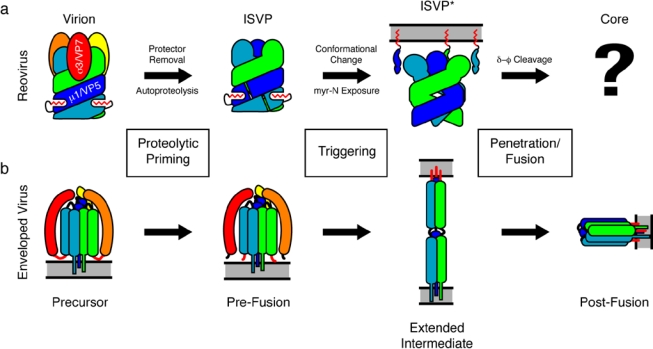
Comparison of the reovirus membrane penetration mechanism with the membrane fusion mechanism of enveloped viruses. **(a)** Cartoon of reovirus membrane penetration. The protection protein (σ3/VP7, warm colors) caps an intertwined trimer of the penetration protein (μ1/VP5, cool colors). The N-terminal myristoylation of the penetration protein is shown in red. The “?” indicates a gap in knowledge about the “post-penetration” conformations of the penetration protein fragments. **(b)** Cartoon of enveloped virus membrane fusion. The functional equivalents to the reovirus protection and penetration protein are colored similarly (e.g., influenza HA_1_ in warm colors and HA_2_ in cool colors), with the fusion peptide indicated in red. Image adapted in part from reference [2].

Based, in part, on the findings reported by Zhang *et al.*, the current model for reovirus membrane penetration suggests the following sequence of events in reovirus entry: Removal of σ3/VP7 by cathepsins promotes autoproteolysis of μ1/VP5, resulting in the meta-stable ISVP [1]. Additional cues trigger dramatic conformational rearrangement of the penetration protein, which passes through the hydrophobic ISVP* state [9] to induce membrane penetration through the release of the myristoylated N-terminal peptide and the ϕ fragment. Although structurally dissimilar, this model for reovirus membrane penetration is conceptually similar to the sequence of events that enveloped virus fusion proteins undergo: A stable precursor is proteolytically cleaved, leading to a meta-stable intermediate, which is triggered to rearrange to an unstable intermediate that drives fusion (or penetration) (Figure 1).

## 3. Achieving High-Resolution Cryo-EM Virus Structures

The aquareovirus ISVP structure is the latest and highest-resolution example of researchers using high-resolution cryo-EM to uncover new findings about viruses that have been studied for decades. At 800 Å in diameter, the aquareovirus ISVP also represents the largest atomic structure solved to date [1]. A 4 Å reconstruction of rotavirus, another member of the *Reoviridae*, was also recently reported [10]. The structure is of the rotavirus double-layered particle (functionally equivalent to the reovirus core) coated with a recombinant version of the major outer capsid protein, VP7. Rotavirus VP7 is the primary virus neutralization target and a critical factor in rotavirus vaccines. The cryo-EM reconstruction of the rotavirus virion revealed that VP7 has a flexible N-terminal arm that extends downward and latches onto the underlying protein (VP6) [10]. Only by high-resolution cryo-EM was this novel interaction discovered; previous lower resolution reconstructions of rotavirus virions, and a crystal structure of VP7 [11], failed to resolve the N-terminal arm. Another example of high-resolution cryo-EM permitting insights into virus biology is provided by a 3.6 Å reconstruction of the bovine papillomavirus capsid [12]. This structure clarified the conformation of the flexible C-terminal arms of the L1 capsid protein, which disulfide-bond among neighboring L1 pentamers. The L1 arms actually loop back to the originating L1 molecule resulting in a “mesh-like” network that stabilizes the virion [12]. Overall, these findings suggest that many stable, geometrically regular virions are suitable for high-resolution cryo-EM analysis. This observation may also extend to well-ordered enveloped viruses, such as those from the *Flaviviridae* family, as a cryo-EM reconstruction of dengue virus at 9.5 Å has already been reported [13].

In addition to a suitable specimen, what is technically required to achieve an atomic resolution structure by cryo-EM? Electron microscopes have certainly become more powerful and feature-rich, yielding better starting images, but much of the advance has been in image processing. Image alignment and refinement of a structure involve iterative manipulation of the collection of particle images. In this process, single virion images are overlaid and compared (“averaged”) to enhance the signal to noise of the starting images. As described for aquareovirus and other structures, appropriate selection and alignment of good-quality particles for use in the refinement process greatly enhances the ability to move into high resolution [1,12]. In other words, the more uniform the specimens, the more images that are used, and the better their alignment, the higher the resolution will be. Newer software allows for greater accuracy of alignment and much more rapid processing of images, which results in a higher-resolution image in a shorter amount of time than was previously possible [14,15]. Additional resolution can come not only from the number of particle images analyzed, but from the degree of symmetry of subunits within a particle. As they were almost structurally identical, the ten quasiequivalent aquareovirus VP5 subunits in each asymmetric unit were averaged, further enhancing the resolution of their structure [1]. Thus, the presence of highly symmetrical subunits within a well-ordered particle or complex may factor into the suitability of a specimen, increasing the potential resolution that can be achieved by cryo-EM.

## 4. Conclusions

Despite the advances in understanding reovirus entry and technical achievement represented in the recent publication by Zhang *et al.*, many biological questions and technical hurdles remain. The structures of the reovirus ISVP* and a “post-penetration” form of μ1 or VP5 have not been determined. These structures would undoubtedly yield further insight into the entry mechanism of nonenveloped viruses. Furthermore, precisely how release of hydrophobic fragments of μ1 or VP5 permit membrane penetration is not known. While there is evidence that fragments of μ1 form pores in membranes, the diameter of these pores appears insufficient to permit the virion core to cross the endocytic membrane [16,17]. In the future, perhaps novel approaches to structural techniques will permit an enhanced capacity to yield information regarding unstable intermediates and biochemically-challenging molecular complexes.

While the averaging of a collection of cryo-EM particle images results in enhancement of those features that are icosahedrally symmetrical, it also has the unfortunate consequence of “averaging out” asymmetrical or disordered regions that may be of significant interest. Most notably, the segmented genome and associated replication enzymes of the *Reoviridae* are not detectable in a meaningful way, even when the symmetrical capsid proteins can be reconstructed at high-resolution [1,3,10]. Observing these features would help to elucidate how *Reoviridae* package and transcribe their genomes from the confines of the virion core. An alternative cryo-EM technique, cryo-EM tomography, in which multiple perspectives of the same particle (not an average of multiple particles) are used to reconstruct an image, may allow researchers to observe asymmetrical and/or rare states of particles (reviewed in [18]). Currently, though, this technique is limited to approximately 25 Å resolution [19].
